# IgG1 glycosylation highlights premature aging in Down syndrome

**DOI:** 10.1111/acel.14167

**Published:** 2024-04-15

**Authors:** Bianca M. M. Streng, Julie Van Coillie, Joanne G. Wildenbeest, Rob S. Binnendijk, Gaby Smits, Gerco den Hartog, Wenjun Wang, Jan Nouta, Federica Linty, Remco Visser, Manfred Wuhrer, Gestur Vidarsson, Louis J. Bont, the PRIDE study group

**Affiliations:** ^1^ Department of Paediatric Infectious Diseases and Immunology, Wilhelmina Children's Hospital University Medical Center Utrecht Utrecht The Netherlands; ^2^ Sanquin Research and Landsteiner Laboratory Amsterdam University Medical Center, University of Amsterdam Amsterdam The Netherlands; ^3^ Department of Biomolecular Mass Spectrometry and Proteomics, Utrecht Institute for Pharmaceutical Sciences and Bijvoet Center for Biomolecular Research Utrecht University Utrecht The Netherlands; ^4^ Centre for Immunology of Infectious Diseases and Vaccines National Institute of Public Health and the Environment Bilthoven The Netherlands; ^5^ Center for Proteomics and Metabolomics, Leiden University Medical Center Leiden The Netherlands

**Keywords:** aging, Down syndrome, glycosylation, immunoglobulin

## Abstract

Down syndrome (DS) is characterized by lowered immune competence and premature aging. We previously showed decreased antibody response following SARS‐CoV‐2 vaccination in adults with DS. IgG1 Fc glycosylation patterns are known to affect the effector function of IgG and are associated with aging. Here, we compare total and anti‐spike (S) IgG1 glycosylation patterns following SARS‐CoV‐2 vaccination in DS and healthy controls (HC). Total and anti‐Spike IgG1 Fc N*‐*glycan glycoprofiles were measured in non‐exposed adults with DS and controls before and after SARS‐CoV‐2 vaccination by liquid chromatography–mass spectrometry (LC–MS) of Fc glycopeptides. We recruited *N* = 44 patients and *N* = 40 controls. We confirmed IgG glycosylation patterns associated with aging in HC and showed premature aging in DS. In DS, we found decreased galactosylation (50.2% vs. 59.0%) and sialylation (6.7% vs. 8.5%) as well as increased fucosylation (97.0% vs. 94.6%) of total IgG. Both cohorts showed similar bisecting GlcNAc of total and anti‐S IgG1 with age. In contrast, anti‐S IgG1 of DS and HC showed highly comparable glycosylation profiles 28 days post vaccination. The IgG1 glycoprofile in DS exhibits strong premature aging. The combination of an early decrease in IgG1 Fc galactosylation and sialylation and increase in fucosylation is predicted to reduce complement activity and decrease FcγRIII binding and subsequent activation, respectively. The altered glycosylation patterns, combined with decreased antibody concentrations, help us understand the susceptibility to severe infections in DS. The effect of premature aging highlights the need for individuals with DS to receive tailored vaccines and/or vaccination schedules.

## Graphical abstract

We found age‐dependent and age‐independent alterations in the IgG1 glycosylation patterns in Down syndrome (DS) compared to a control group. The altered glycosylation patterns, combined with decreased antibody concentrations, help us understand the susceptibility to severe infections in DS.

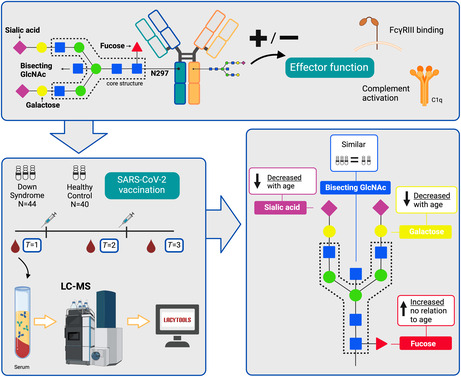

**Abbreviations**
ADCC: antibody‐dependent cellular cytotoxicity
ASD: atrial septum defect
AVSD: atrioventricular septum defect
DM: diabetes mellitus
DS: Down syndrome
GlcNAc: N‐acetylglucosamine
HC: healthy control
IgG: immunoglobulin G
LC‐MS: liquid chromatography‐mass spectrometry
MIA: multiplex immunoassay
N: nucleocapsid protein
RTI: respiratory tract infection
S: spike protein
SARS‐CoV‐2: severe acute respiratory syndrome coronavirus 2
TGA: transposition of the great arteries
VSD: ventricular septum defect

## 1 INTRODUCTION

Down syndrome (DS), or Trisomy 21, is the most common chromosomal disorder worldwide (Chen et al., 2022; Van Gameren‐Oosterom et al., 2012). DS is characterized by a wide range of deficits, such as moderate to severe intellectual disability, multiple congenital abnormalities, and comorbidities such as hypothyroidism, celiac disease, and diabetes mellitus (Bergholdt et al., 2006; Bonamico et al., 2001; Borstlap et al., 2011). During their lifetime, individuals with DS are susceptible to respiratory tract infections (RTIs), which lead to high numbers of hospital admissions and mortality (Beckhaus & Castro‐Rodriguez, 2018; Bloemers, Broers, et al., 2010; Manikam et al., 2020). This increased susceptibility might be due to anatomical differences, comorbid conditions (McDowell & Craven, 2011), and alterations in the immune system, such as lower absolute numbers of circulating B and T cells (Bloemers, van Bleek, et al., 2010; De Hingh et al., 2005) and premature aging or immunosenescence of the immune system (Gensous et al., 2020; Trotta et al., 2011).

**FIGURE 1:**
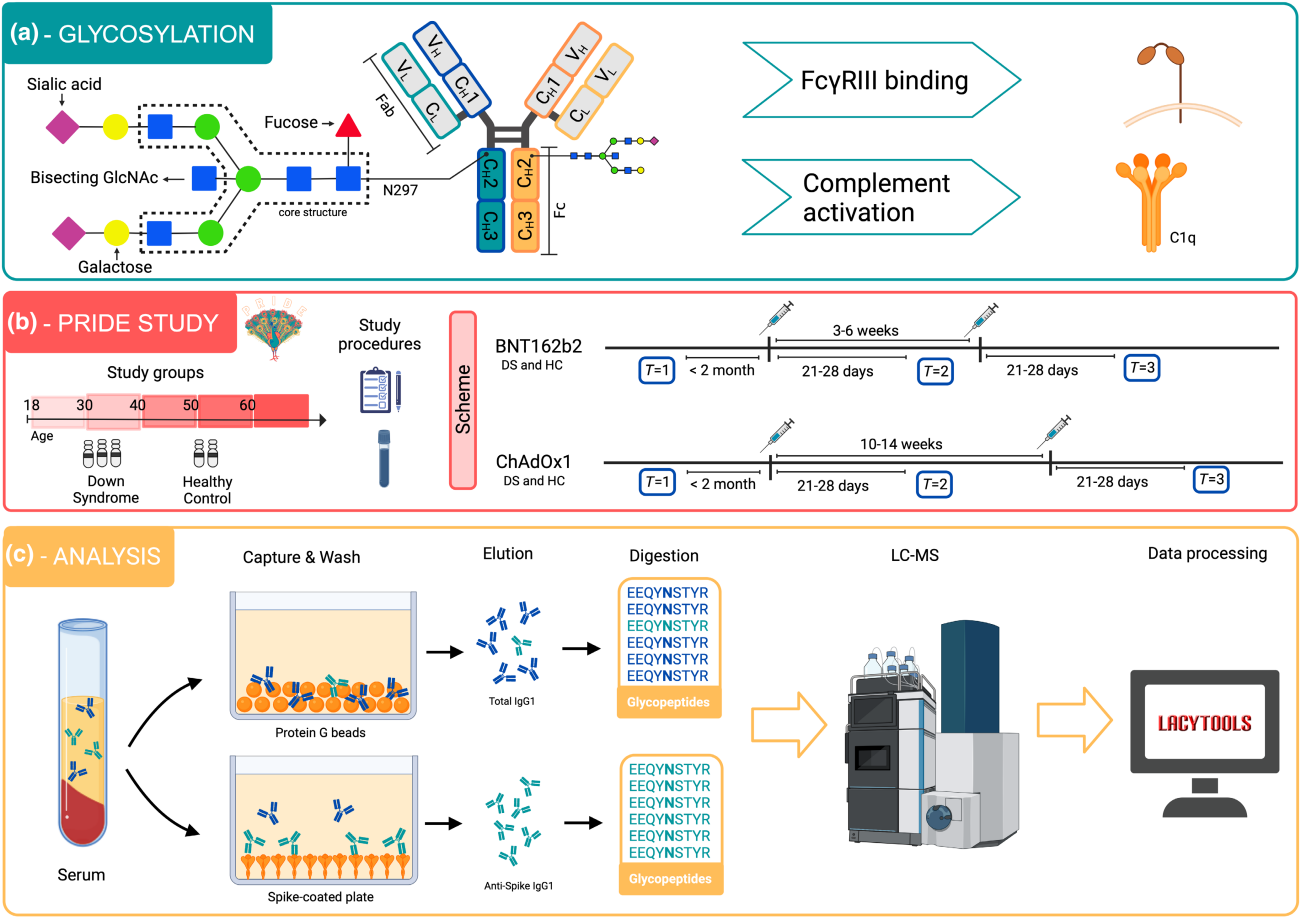
Overview of the PRIDE study and analysis. (a) Schematic representation of antibody Fc N‐glycosylation and effector functions. (b) Overview of PRIDE study and timing of sampling. (c) Schematic representation of the antibody glycomics workflow. *Created with* BioRender.com.

Premature aging in DS is reflected in the well‐known early onset of Alzheimer's disease (Veteleanu et al., 2023; Wiseman et al., 2015), but it they also present earlier age‐related reduction of the regenerative capacity of tissues (Kusters et al., 2011; Xu et al., 2022). Underlying mechanisms have been studied (Bhattacharya et al., 2020; Xu et al., 2022), but causative mechanisms are still unknown (Kusters et al., 2011). Immunoglobulin G (IgG) is a glycoprotein with a conserved *N*‐linked glycan at its Fc domain at position N297 (Figure 1) and serves as a biomarker for chronological and biological aging (Krištić et al., 2014). This *N*‐glycan consists of a core structure, which can further be elongated by bisected N‐acetylglucosamine (GlcNAc), one or two galactoses, one or two sialic acids, and/or fucose. The IgG Fc *N*‐glycan influences IgG effector functions (Golay et al., 2022). Functionally, the bisecting GlcNAc is not known to affect IgG effector functions (Dekkers et al., 2017; van Osch et al., 2021). In contrast, core fucosylation and galactosylation/sialylation influence Fc gamma receptor III (FcγRIII) binding and IgG complement activation respectively. Afucosylated IgG has an increased affinity (up to 40 times) to the FcγRIII receptor family, enhancing the activation of myeloid and NK cells, and subsequent antibody‐dependent cellular cytotoxicity (ADCC) and phagocytosis (Golay et al., 2022; Vidarsson et al., 2014). In healthy conditions, approximately 94% of all IgG‐Fc N‐glycans are fucosylated (Wuhrer et al., 2007). Unlike IgG‐Fc fucosylation, Fc galactosylation and sialylation levels are highly variable in healthy individuals and both decline with age (Baković et al., 2013; Gudelj et al., 2018; Krištić et al., 2014).

In healthy individuals, SARS‐CoV‐2 vaccination induces a transient increase in anti‐S IgG Fc afucosylation (a lack of fucose), galactosylation, and sialylation. While there are indications that the glycosylation pattern in DS is altered (Borelli et al., 2015; Cindric et al., 2021; Murray et al., 2023), no studies of anti‐S or antigen‐specific IgG Fc glycosylation in DS have been performed. In this study, we aimed to gain insight into the total and anti‐S IgG‐Fc glycosylation pattern upon SARS‐CoV‐2 vaccination in individuals with DS and evaluated the effect of aging by comparison to an age‐matched healthy control (HC) group.

## 2 METHODS

### 2.1 Study design and participants

The Prospective Monitoring of Antibody Response Following COVID‐19 Vaccination in Patients with Down Syndrome (PRIDE) study was previously described (Streng et al., 2022). The PRIDE study is a prospective observational cohort study in the Netherlands comparing the immune response of individuals with DS to HC after SARS‐CoV‐2 vaccination. Exclusion criteria included receipt of an organ transplant, active malignancy, treatment for malignancy in the past 3 months, and human immunodeficiency virus infection. HCs were also excluded for any condition needing regular visits to a healthcare provider. Adult participants with previous exposure to SARS‐CoV‐2 were excluded when they had anti‐S IgG at *T* = 1 (>10.08 BAU/mL) or anti‐N IgG concentration at any timepoint (>14.3 BAU/mL) (van den Hoogen et al., 2021). All participants received either BNT162b2 (Pfizer BioNTech) or ChAdOx1 (AstraZeneca) routine vaccination and had available serology at least two timepoints. The University Medical Centre Utrecht medical research ethics committee (NL76336.041.21) approved this study and all participants and/or legal representatives provided written informed consent before inclusion in the PRIDE study.

### 2.2 Study procedures

Participants received two doses of either BNT162b2 (Pfizer/BioNTech, 3–6 week interval) or ChAdOx1 (AstraZeneca, 10–14 week interval), as part of the Dutch national immunization program. Blood samples were collected at three timepoints: *T* = 1, <2 months before the first vaccination, *T* = 2 at 21–28 days after the first vaccination, and *T* = 3, 28 days (range, 21–42 days) after the second vaccination.

### 2.3 IgG concentration analysis by multiplex immunoassay (MIA)

SARS‐CoV‐2 IgG concentrations against the spike protein (S) and nucleocapsid protein (N) were measured by multiplex immunoassay, as previously described (Den Hartog et al., 2020, 2021; Geers et al., 2021). Concentrations were reported as binding antibody units per milliliter (BAU/mL).

### 2.4 IgG fc glycosylation analysis by mass spectrometry

Anti‐S IgG Abs were affinity‐captured, as described elsewhere (Brouwer et al., 2020; Larsen et al., 2021; Van Coillie, Pongracz, Rahmöller, et al., 2023). In brief, spike protein‐coated plates (Brouwer et al., 2020; Larsen et al., 2021) were incubated with plasma or serum from donors followed by a formic acid elution step. Total IgG Abs were affinity‐captured from plasma or sera using a Protein G AssayMAP Cartridge Rack on the Bravo (Agilent Technologies, Santa Clara, CA), as described elsewhere (Larsen et al., 2021; Van Coillie, Pongracz, Rahmöller, et al., 2023). Both anti‐S and total IgG eluates were subjected to tryptic cleavage followed by LC–MS analysis, as described previously (Larsen et al., 2021; Van Coillie, Pongracz, Rahmöller, et al., 2023).

### 2.5 LC–MS data processing

LC–MS spectra were analyzed using the in‐house developed software LaCyTools (Jansen et al., 2016). Alignment was performed based on the average retention time of at least three highly abundant glycoforms. The analyte list for targeted extraction of the 2^+^ and 3^+^ charge states was based on manual annotation as well as on literature reports (Pucić et al., 2011). The inclusion of an analyte was based on quality criteria: signal‐to‐noise higher than 9, isotopic pattern quality less than 25% deviation from the theoretical isotopic pattern, and mass error within ±20 parts per million range (Table S1). The glycosylation traits IgG1 Fc bisection, galactosylation, sialylation, and fucosylation were calculated by normalizing the relative intensity of each glycopeptide species to the sum of their total areas (Table S2), analogously to previous reports (Brouwer et al., 2020; Larsen et al., 2021).

### 2.6 Statistical analysis

Baseline characteristics are presented as absolute numbers and percentages, continuous data presented as mean with standard deviation (SD). A Chi‐squared test was used comparing categorical parameters, unpaired or paired Student's *t* test for normally distributed continuous parameters and a Mann–Whitney U test or Wilcoxon signed‐rank test in case of not normally distributed continuous parameters. A Pearson's test was used for correlations. Due to the small number of participants, we did not conduct a multivariate analysis. Statistical analyses were performed in IBM SPSS Statistics 28.0.1.1 software (IBM, Armonk, New York) and graphs were produced using GraphPad Prism version 10.0.1 for MacOS. A *p* value of 0.05 was considered statistically significant.

## 3 RESULTS

### 3.1 Study cohort

**FIGURE 2:**
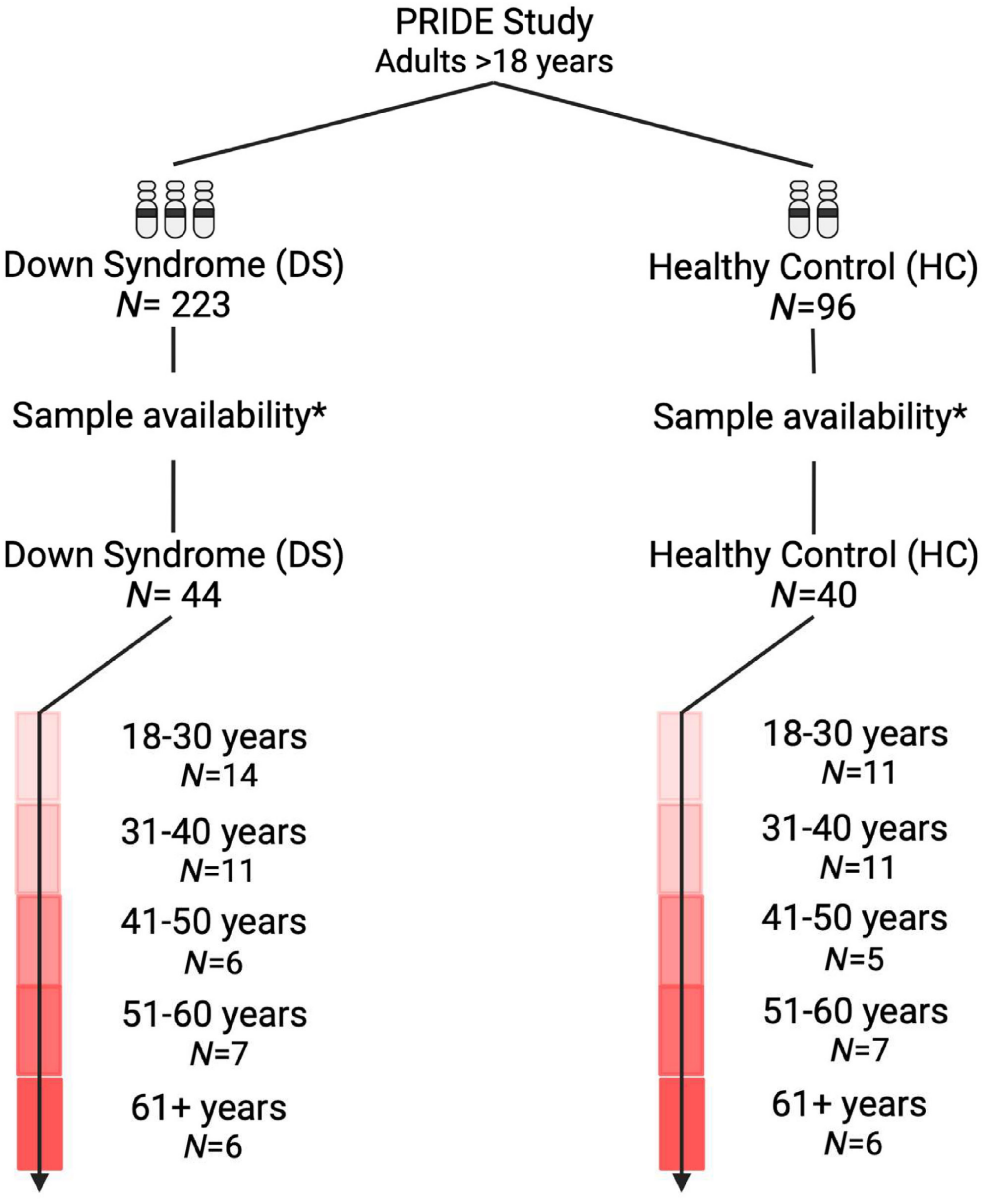
Flow diagram and selection. Samples were selected based on the age of the participant and the availability of samples at timepoints *T* = 1, *T* = 2, and *T* = 3. *Included participants in this analysis should at least have samples available for *T* = 2 and *T* = 3. *Created with* BioRender.com.

Vaccine‐induced responses were compared between vaccinees with DS (*N* = 44) and HC (*N* = 40), routinely vaccinated with either the mRNA vaccine BNT162b2 or adenoviral‐based ChAdOx1 vaccine (Figure 2; Table 1). Vaccinees were sampled longitudinally at three timepoints, before the first vaccination (*T* = 1), ±28 days after first vaccination (*T* = 2) and ± 28 days after second vaccination (*T* = 3). The second vaccine dose was administered 3–6 or 10‐14 weeks after the first dose for BNT162b2 or ChAdOx1, respectively. (Figure 1).

**TABLE 1:** Baseline characteristics.

	DS (*N* = 44)	HC (*N* = 40)
Age [mean, SD]	40 [16]	43 [15]
Male gender [*n*, %]	24 [56%]	15 [38%]
Ethnicity		
North West European [*n*, %]	40 [91%]	37 [93%]
Smoking [*n*, %]	1 [2%]	4 [11%]
SARS‐CoV‐2 Vaccine type		
BNT162b2 [*n*, %]	28 [64%]	22 [55%]
ChAdOx1 [*n*, %]	16 [36%]	18 [45%]
Medical history		
Congenital heart disease^a^ [*n*, %]	9 [21%]	0
Thyroid disease [*n*, %]	20 [46%]	2 [5%]
Celiac disease [*n*, %]	0	0
Diabetes mellitus^b^ [*n*, %]	0	0
Other primary or secondary immunodeficiency^c^ [*n*, %]	2 [5%]	0
Immunosuppressive medication [*n*, %] (LCI‐RIVM, 2023)	2 [5%]	0

^a^ All congenital heart diseases including atrioventricular septum defect (AVSD), atrial septum defect (ASD), ventricular septum defect (VSD), Tetralogy of Fallot, Transposition of the great arteries (TGA).

^b^ Including DM type 1 and type 2.

^c^ Both secondary immunodeficiency due to immunosuppressive medication.

### 3.2 Accelerated aging of total IgG1 fc N‐Glycosylation in Down syndrome

**FIGURE 3:**
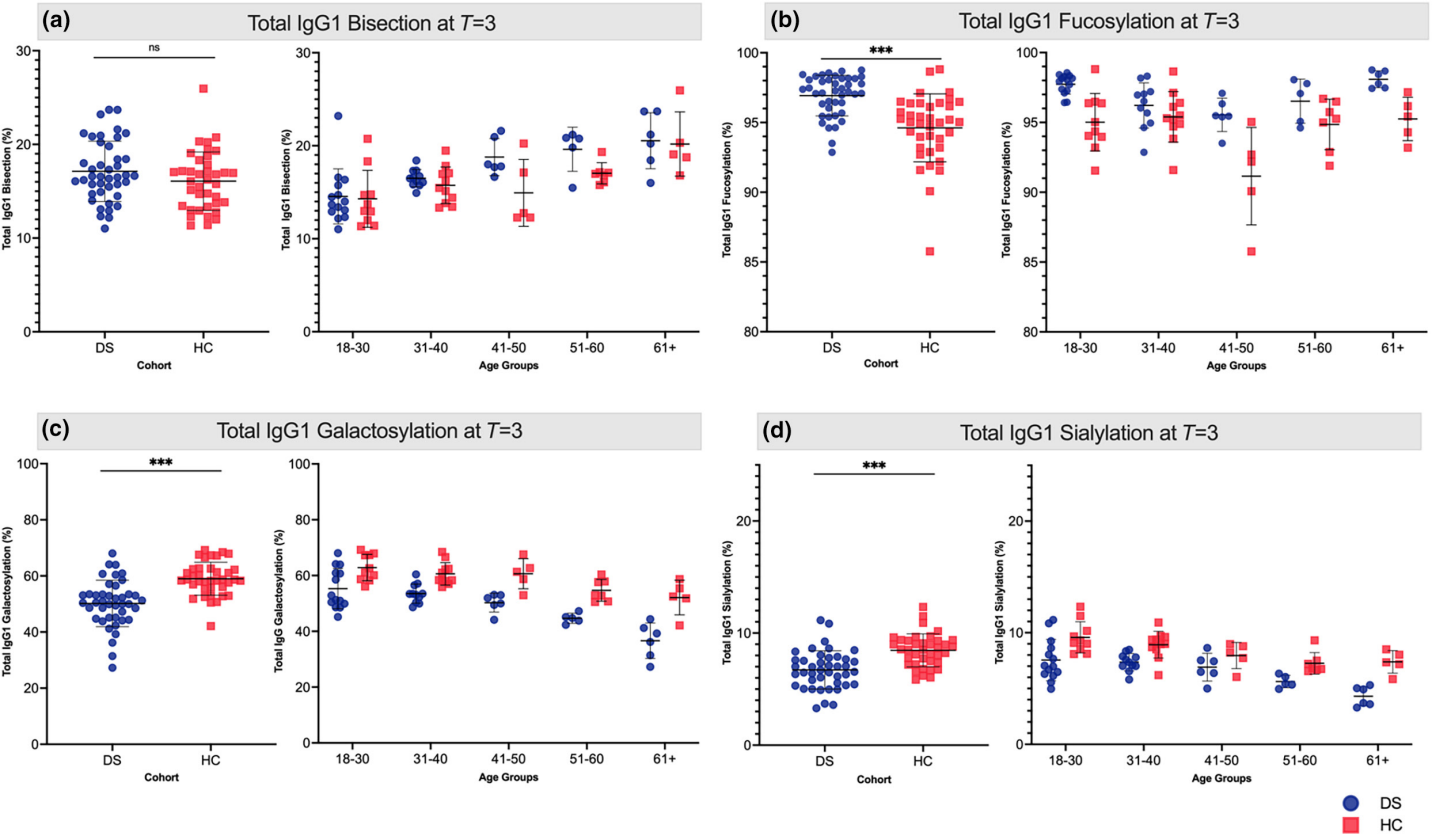
Total IgG1 Fc N‐glycosylation in DS and HC at *T* = 3. Total IgG1 Fc bisection, fucosylation, galactosylation, and sialylation at *T* = 3 (±28 days after second vaccination) for both individuals with Down syndrome (DS, blue circle) and healthy controls (HC, red square). (a) DS correlation to age (Pearson R 0.695, *p*‐value <0.001); (b) DS correlation to age (Pearson *R* = −0.109, *p‐*value = 0.491); (c) DS correlation to age (Pearson *R* = −0.109, *p*‐value = 0.491); (d) DS correlation to age (Pearson *R* = −0.610, *p*‐value <0.001). Student's *t* test was used to compare DS and HC with ns, not significant; *p‐value <0.05; **p‐value <0.01; ***p‐value <0.001.

As the anti‐S response is mostly of the IgG1 subclass (Espino et al., 2024), we focused our efforts on IgG1‐Fc glycosylation. As expected, total IgG1 Fc fucosylation, galactosylation, sialylation, and bisection were stable over time (Figure S1). There was substantial variation in glycosylation patterns between individuals. Total IgG1 bisection was similar for DS and HC and increased with age in both DS and HC. IgG1 Fc fucosylation was higher for DS than for HC, independent of age. Both galactosylation and sialylation were lower in DS than in HC and showed an age‐dependent decrease for both groups (Figure 3a–d). The average levels of total IgG1 galactosylation and sialylation in DS corresponded to those of 28 or 30 year older HC participants, respectively (Figure S2).

### 3.3 Anti‐S1 antibody concentrations following SARS‐CoV‐2 vaccination

Anti‐Spike S1 subunit antibody concentrations were measured at the three timepoints. None of the participants showed anti‐S1 antibodies before the first dose (*T* = 1) (Figure S3) or anti‐nucleocapsid (N) antibodies at any timepoint. (Figure S4). As previously shown (Streng et al., 2022), participants with DS showed decreased anti‐S1 IgG concentrations at *T* = 3 compared to HC (Figure S3). This was significant for the participants receiving BNT162b2, for the smaller cohort receiving ChAdOx1 this difference could not be evaluated given the low numbers of samples (Figure S3). Also, the anti‐S antibody concentration following mRNA vaccination showed an age‐dependent decreased antibody response. No such effect was observed in HC (Figure S3).

### 3.4 Distinct anti‐S IgG1 fc N‐glycosylation following SARS‐CoV‐2 vaccination

Anti‐S IgG1 showed increased Fc fucosylation, galactosylation, and sialylation, but decreased Fc bisection compared to total IgG1 across both cohorts. (Figure S5). Anti‐S IgG1 Fc glycosylation fluctuated over time across all cohorts with an increase in time at *T* = 3 for fucosylation and bisection. In contrast to fucosylation and bisection, galactosylation, and anti‐S IgG decreased in time, whereas sialylation remained stable (Figure S6).

### 3.5 Accelerated aging in DS anti‐S IgG1 fc N‐glycosylation signature

**FIGURE 4:**
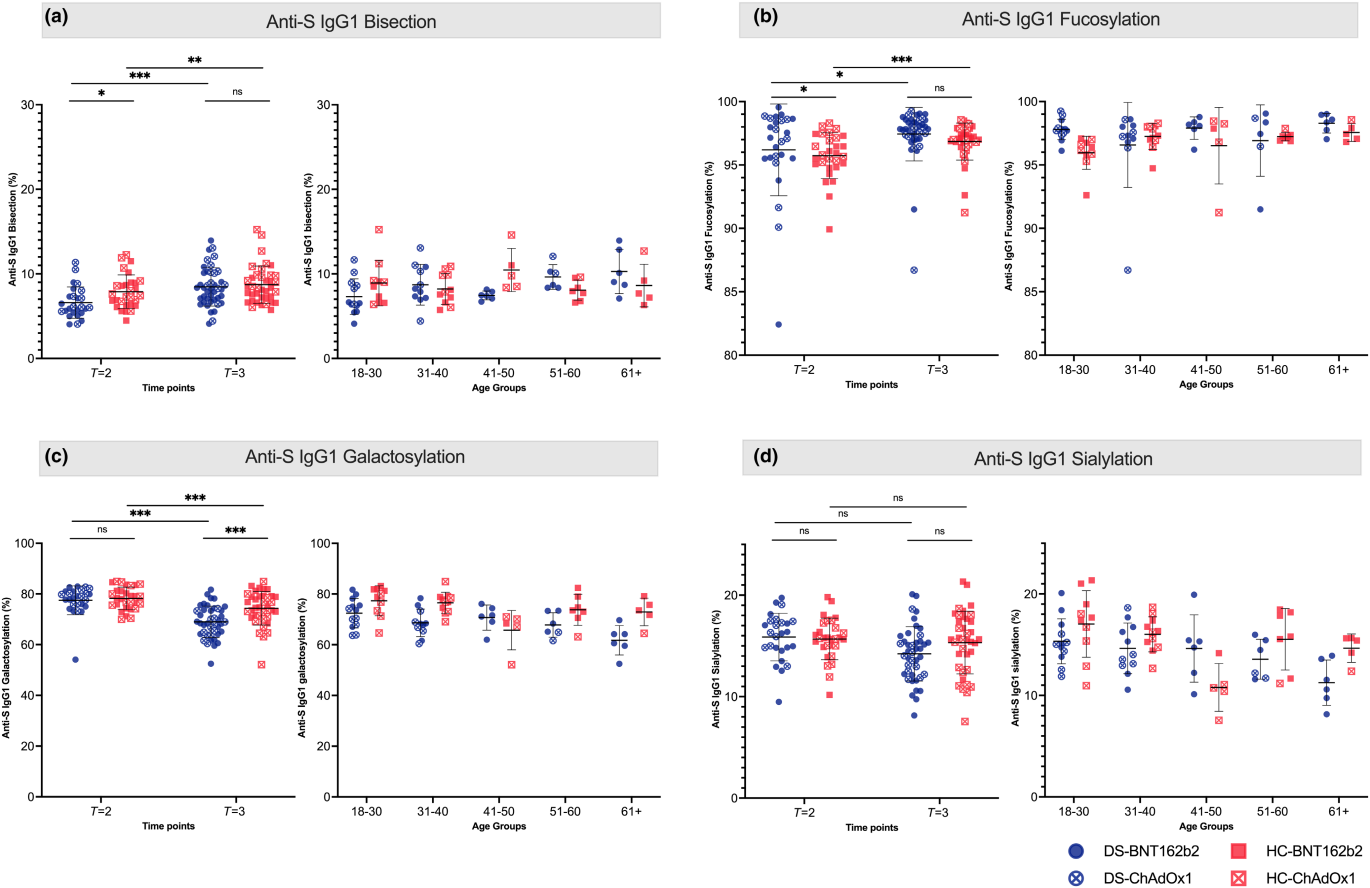
Anti‐S IgG1 Fc N‐glycosylation in DS and HC. Anti‐S IgG1 Fc fucosylation (b), galactosylation (c), bisection (a), and sialylation (d) at *T* = 2 (±28 days after second vaccination) and *T* = 3 (±28 days after second vaccination) for individuals with Down syndrome (DS, blue circle) and healthy controls (HCs, red square) vaccinated with either the BNT162b2 (solid) or the ChAdOx1 (empty) vaccine. (c) DS correlation to age (Pearson *R* = −0.435, *p*‐value = 0.004. (d) DS correlation to age (Pearson *R* = −0.441, *p*‐value = 0.003). Differences were statistically tested with Student's *t* test, paired samples *t* test, Mann–Whitney U test, or Wilcoxon signed‐rank test, depending on normal the distribution of samples with ns, not significant; *p‐value <0.05; **p‐value <0.01; ***p‐value <0.001.

The effect of aging in the glycosylation pattern of specific IgG1 was similar to total IgG1. We observed that the general decrease in IgG1 galactosylation according to age starts earlier in DS as seen in total IgG1. (Figure 4a–d). Similarly, an early decrease in anti‐S IgG1 sialylation with increasing age is seen in DS. The average levels of anti‐S IgG1 galactosylation in DS correspond with 36 to 17 year older HC participants (Figure S2). In the fucosylation and bisection of anti‐S IgG1, no effects of aging were seen.

## 4 DISCUSSION

**FIGURE 5:**
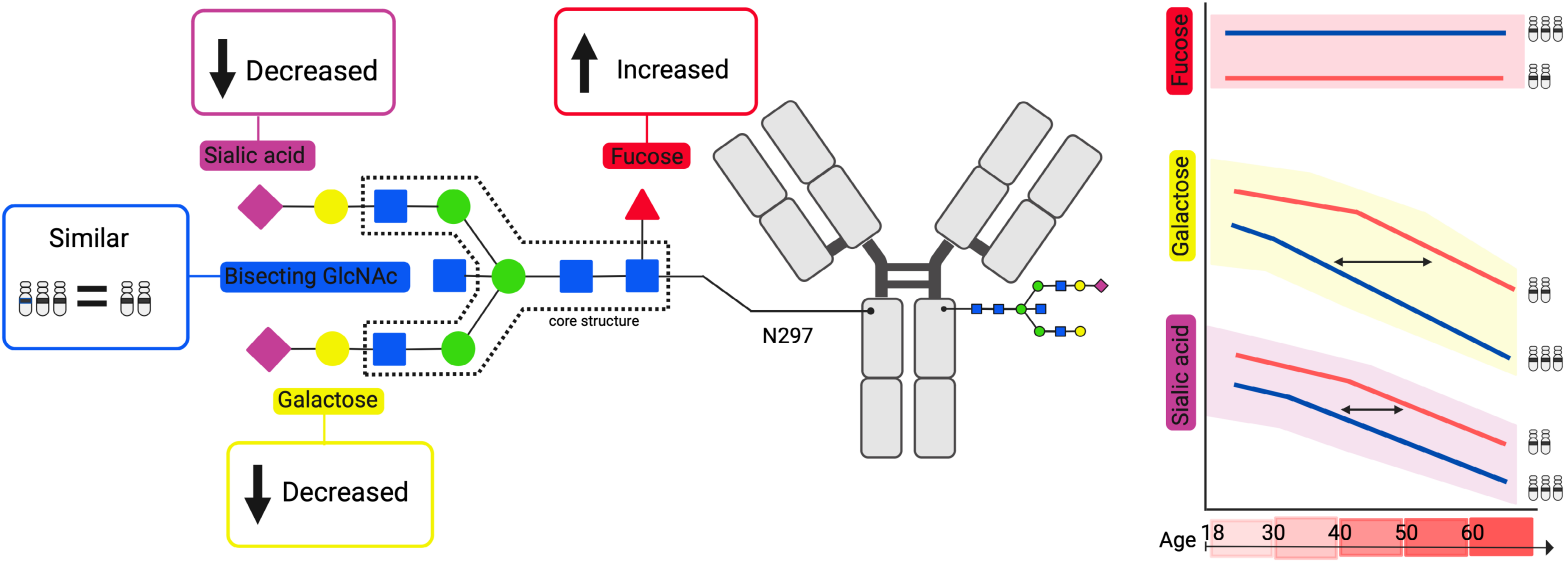
IgG1 glycosylation highlights premature aging in Down syndrome. IgG1 fucosylation is increased in DS compared with HC, with no relation to age. IgG1 galactosylation and sialylation, however, are decreased in DS. Both show a premature decline with increasing age, comparing them to HC.

In this study, we aimed to gain insight into the total and anti‐S IgG1 Fc glycosylation pattern upon SARS‐CoV‐2 vaccination in individuals with DS and evaluate the effect of aging on this pattern. Our results show that individuals with DS show both age‐dependent and age‐independent alterations in the glycosylation trait of both total and anti‐S IgG1 (Figure 5). This is first reflected in an early decrease in total and anti‐S IgG1 galactosylation and sialylation leading to comparable values measured in HCs of ±30 years older. Second, we found an age‐independent increase in total and anti‐S IgG1 fucosylation.

Antibodies have a conserved N‐glycan linked in their Fc domain, influencing IgG effector functions. Age, sex, and other demographic characteristics are known to influence IgG Fc glycosylation. For example, total IgG Fc galactosylation and sialylation, decline with age (Baković et al., 2013; Krištić et al., 2014), whereas bisection is described to increase with age (Gudelj et al., 2018).

In this study, in line with literature, we observed an increase in total IgG1 Fc bisection, with increasing age in both DS and HC (Gudelj et al., 2018). Anti‐S IgG1 Fc bisection was increased compared to total IgG at both *T* = 2 and *T* = 3, in line with literature (Chakraborty, Gonzalez, Edwards, et al., 2021; Chakraborty, Gonzalez, Sievers, et al., 2021; Larsen et al., 2021; Van Coillie, Pongracz, Rahmöller, et al., 2023; Van Coillie, Pongracz, Šuštić, et al., 2023). IgG Fc bisection is not known to directly influence IgG effector functions such as complement or Fc receptor binding and subsequent activation (Dekkers et al., 2017; van Osch et al., 2021).

Total IgG Fc fucosylation levels are high during childhood and slightly decreases during adolescence, after which it remains stable (Oosterhoff et al., 2022). Interestingly, here, we found total IgG Fc fucosylation in DS to be significantly higher compared to HC, independent of age. A decrease in antigen‐specific Fc fucosylation is seen in some immune responses, such as against enveloped viral proteins (Larsen et al., 2021; Oosterhoff et al., 2022). Furthermore, a decrease in anti‐S IgG1 Fc fucosylation is observed after both SARS‐CoV‐2 infection and mRNA and adenovirus‐based vaccination (Chakraborty, Gonzalez, Edwards, et al., 2021; Chakraborty, Gonzalez, Sievers, et al., 2021; Larsen et al., 2021; Pongracz et al., 2022; Siekman et al., 2022; Van Coillie, Pongracz, Rahmöller, et al., 2023; Van Coillie, Pongracz, Šuštić, et al., 2023). Antibodies lacking core fucose have an increased IgG binding to FcγRIIIa/b of up to 40‐fold (Golay et al., 2022; Vidarsson et al., 2014). This can lead to increased cytokine production and/or ADCC. Furthermore, IgG1 Fc core afucosylation has been shown to directly correlate with SARS‐CoV‐2 disease severity (Chakraborty, Gonzalez, Edwards, et al., 2021; Hoepel et al., 2021; Larsen et al., 2021). For anti‐S IgG1 Fc fucosylation we observed a transient decrease in the HC group, in line with literature (Chakraborty, Gonzalez, Edwards, et al., 2021; Chakraborty, Gonzalez, Sievers, et al., 2021; Larsen et al., 2021; Van Coillie, Pongracz, Rahmöller, et al., 2023; Van Coillie, Pongracz, Šuštić, et al., 2023). Interestingly, this transient decrease at *T* = 2 was not observed in the DS group, in line with their elevated total IgG1 Fc fucosylation.

In all individuals, anti‐S IgG1 Fc fucosylation levels increased in time and reached levels comparable to total IgG levels at *T* = 3. The observed high anti‐S IgG Fc fucosylation levels in the DS group is hypothesized to give less FcγRIIIa/b activation and hence decreased cytokine production and ADCC (Oosterhoff et al., 2022). Therefore, it might potentially be protecting against exaggerated immune responses after SARS‐CoV‐2 infection. However, afucosylated IgG may also have a protective function by enhanced clearance through monocytes, lowering the viral load and inflammation.

Total IgG1 Fc galactosylation and sialylation was decreased in DS compared to HC, but declined in both cohorts, in line with literature (Baković et al., 2013; Gudelj et al., 2018; Krištić et al., 2014). However, individuals with DS show an early decrease in these glycan traits compared to age‐matched HC (Baković et al., 2013; Borelli et al., 2015; Cindric et al., 2021; Krištić et al., 2014). This trend is in line with the general premature aging in observed in DS (Horvath et al., 2015; Obeid et al., 2016; Xu et al., 2022). In contrast to fucosylation, significantly increased galactosylation and sialylation is seen upon SARS‐CoV‐2 vaccination and natural infection (Chakraborty, Gonzalez, Edwards, et al., 2021; Chakraborty, Gonzalez, Sievers, et al., 2021; Pongracz et al., 2022; Van Coillie, Pongracz, Rahmöller, et al., 2023; Van Coillie, Pongracz, Šuštić, et al., 2023). In our study, both DS and HC showed increased anti‐S IgG1 Fc galactosylation and sialylation, compared to total IgG. Functionally, increased IgG galactosylation enhances IgG hexamerization which increases complement component 1q (C1q) docking and subsequent complement activation (van Osch et al., 2021; Wei et al., 2021), indicating that individuals with DS are less protected through complement compared to HC.

This study has some limitations. Although we present a relatively large number of participants in all age groups of the DS and HC cohort, we were not able to perform a multivariate analysis to correct for known confounders or investigate correlations. In addition, due to the objective study design and changing vaccination strategy in the SARS‐CoV‐2 pandemic, not all series of samples per participant were complete and available for analysis. Furthermore, although these results might point toward decreased function of the IgG derived immunity in DS, we did not perform functional assays to confirm this.

In summary, our data show a signature of premature aging of IgG1 Fc glycosylation in individuals with Down syndrome compared to HCs. This is reflected in an age‐dependent decrease in both total and anti‐S IgG1 Fc galactosylation and sialylation. We furthermore observed an age‐independent increase of both total and anti‐S IgG1 Fc fucosylation in DS compared to HC. This outcome leads to the conclusion that patients with DS have IgG antibodies that are less capable of complement activation and Fc‐receptor engagement. These findings, in combination with decreased anti‐S antibody concentrations following vaccination, help us understand the high prevalence of RTIs and auto‐immunity in DS. Our findings further highlight the need for an older adult approach of individuals with DS with for instance a vaccine regimen tailored specifically to individuals with DS for enhanced protective immunity in this population.

## AUTHOR CONTRIBUTIONS

B.M.M. Streng, J. Van Coillie, G. Vidarsson J.G. Wildenbeest, and L.J. Bont contributed to the conceptualization and design of the study and/or sample and data collection. B.M.M. Streng, J. Van Coillie, G. Vidarsson, R.S. Binnendijk, G. Smits, G. den Hartog, W. Wang, J. Nouta, F. Linty, R. Visser, M. Wuhrer, and L.J. Bont were involved in laboratory experiments and/or analysis and interpretation of collected data. All authors contributed to writing, review, and editing of the report. The corresponding author had full access to all data and the final responsibility to submit for publication.

## FUNDING INFORMATION

This work was supported by ZonMw, The Netherlands Organization for Health Research and Development grant 10430072010004 (LB). Landsteiner foundation for Blood Transfusion Research (LSBR) grants 1721 and 1908 (GV) ZonMW COVID‐19 grant 10430012010021 (GV).

## CONFLICT OF INTEREST STATEMENT

LJB has regular interaction with pharmaceutical and other industrial partners. He has not received personal fees or other personal benefits. UMCU has received major funding (>€100,000 per industrial partner) for investigator‐initiated studies from AbbVie, MedImmune, AstraZeneca, Sanofi, Janssen, Pfizer, MSD, and MeMed Diagnostics. UMCU has received major funding for the RSV GOLD study from the Bill & Melinda Gates Foundation. UMCU has received major funding as part of the public private partnership IMI‐funded RESCEU and PROMISE projects with partners GSK, Novavax, Janssen, AstraZeneca, Pfizer, and Sanofi. UMCU has received major funding from Julius Clinical for participating in clinical studies sponsored by MedImmune and Pfizer. UMCU received minor funding (€1000–25,000 per industrial partner) for consultation and invited lectures by AbbVie, MedImmune, Ablynx, Bavaria Nordic, mAbXience, GSK, Novavax, Pfizer, Moderna, AstraZeneca, MSD, Sanofi, Genzyme, and Janssen. LJB is the founding chairman of the ReSViNET Foundation. JGW has been an investigator for clinical trials sponsored by pharmaceutical companies including AstraZeneca, Merck, Pfizer, Sanofi, and Janssen. All funds have been paid to UMCU. JGW participated in the advisory board of Janssen and Sanofi with fees paid to UMCU. All other authors declare they have no conflicts of interest.

## Supporting information


Appendix S1

Appendix S2

## Data Availability

The data generated and/or analyzed during this study are available upon request from the corresponding author.
