# Supplementary material for: IgG1 glycosylation highlights premature aging in Down syndrome
Source: Aging Cell. 2024 Apr 15;23(7):e14167. doi: 10.1111/acel.14167 (PMC11258452; doi:10.1111/acel.14167)
Supplement: Supplementary file 1 — Appendix S1 [file ACEL-23-e14167-s002.pdf]

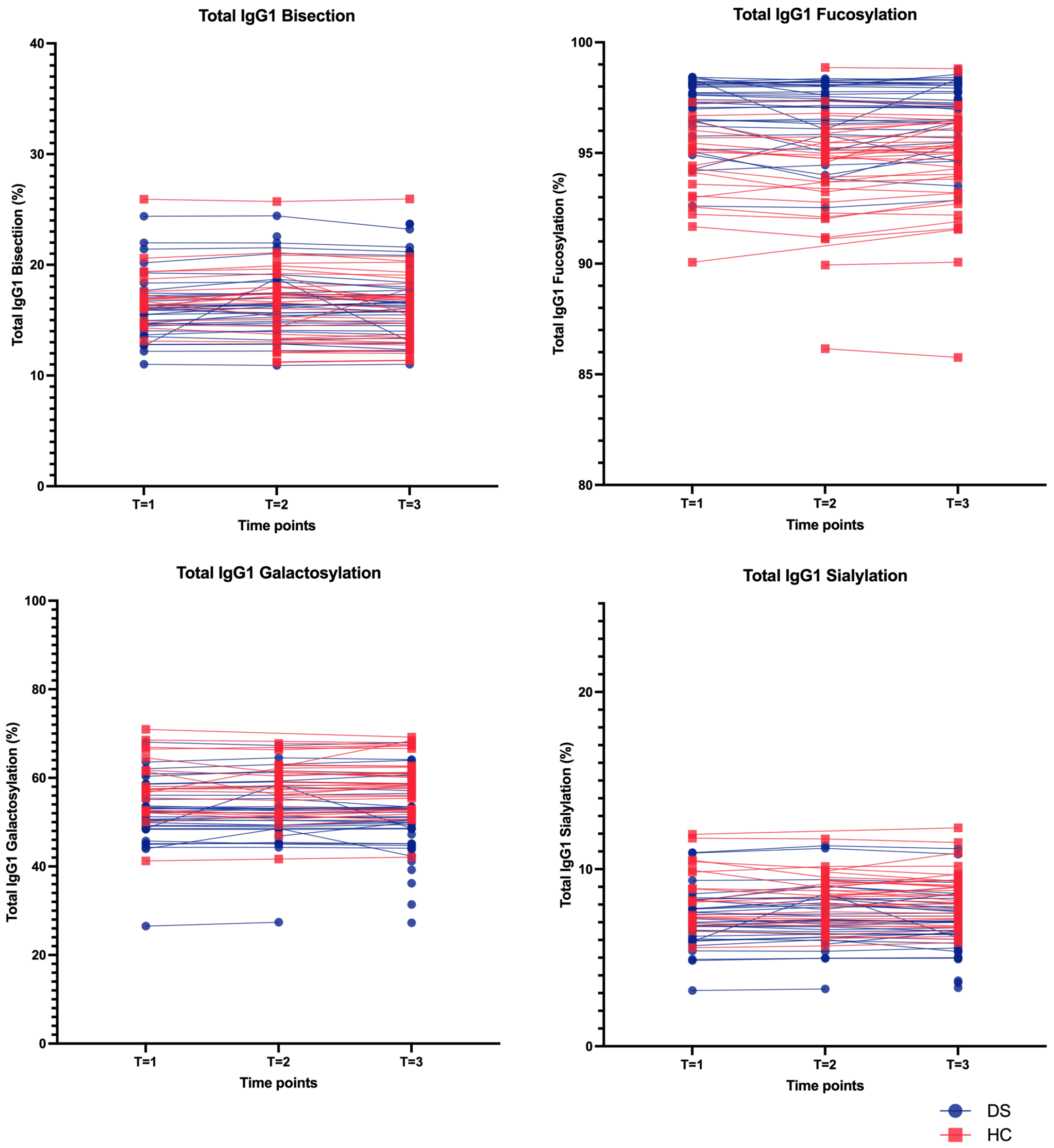

Supplemental Figure 1 - Total IgG1 Fc N-glycosylation in time

Total IgG1 glycosylation at T=1 (before vaccination), T=2 (±28 days after 1st vaccination) and T=3 (±28 days after 2nd vaccination) for individuals with Down Syndrome (DS, blue circle) and Healthy Control (HC, red square).

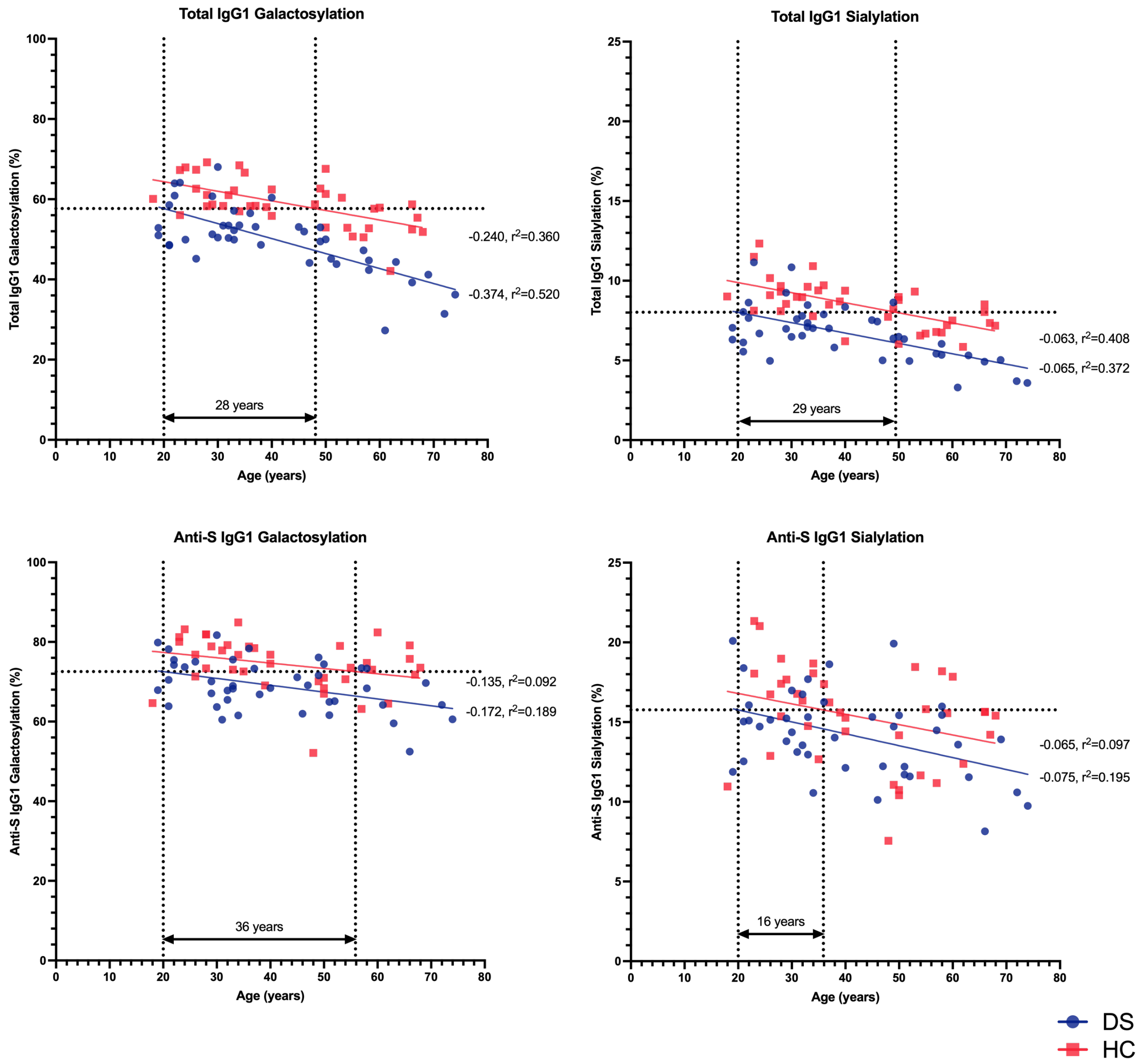

Supplemental Figure 2. Influence of age on total and IgG1 Fc galactosylation and sialylation at T=3

Influence of age on total and anti-S IgG1 Fc in DS and HC by simple linear regression model with slope and R^2.

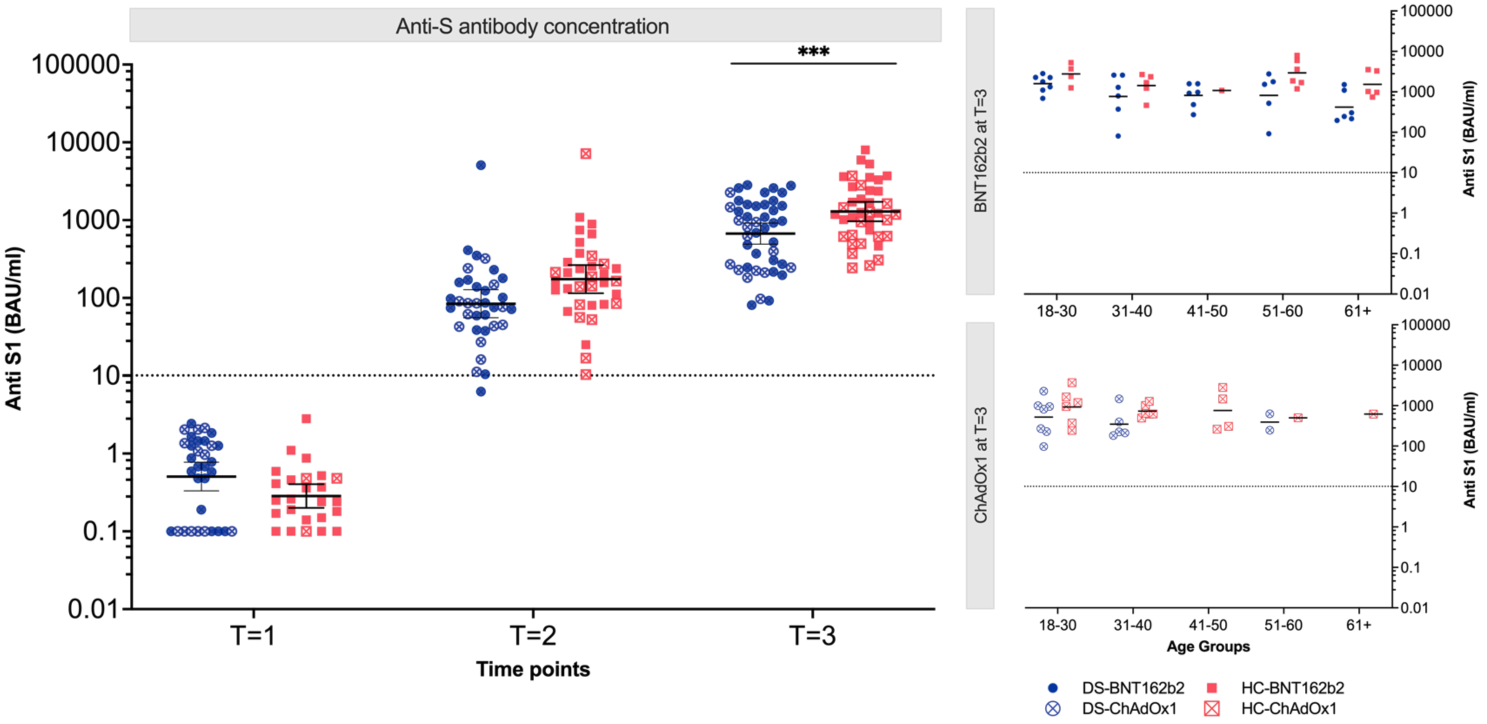

Supplemental figure 3 – Anti-Spike antibody concentration following SARS-CoV-2 vaccination

Anti-spike subunit 1 (S1) antibody concentration in Binding Antibody Units (BAU) per ml at T=1 (before vaccination), T=2 (±28 days after 2nd vaccination) and T=3 (±28 days after 2nd vaccination) for individuals with Down Syndrome (DS, blue circle) and healthy controls (HC, red square) vaccinated with either the BNT162b2 (solid) or ChAdOx1 (empty) vaccine, as previously published (1).

Students T- test was used to compare DS and HC with ns; not significant, *; *p*-value <0.05, **; *p*-value < 0.01, ***; *p*-value < 0.001.

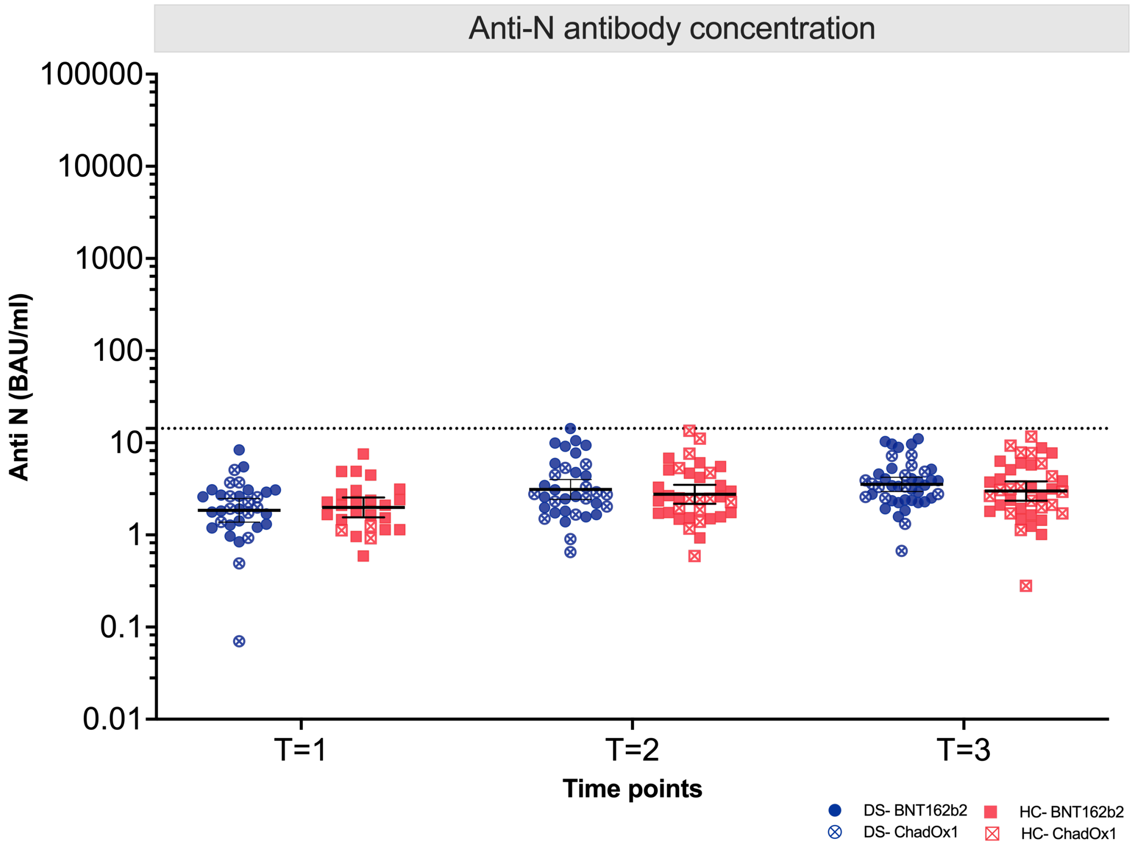

Supplemental figure 4 – anti-N antibody concentration

Anti-N antibody concentrations at T=1 (before vaccination), T=2 (±28 days after 2nd vaccination) and T=3 (±28 days after 2nd vaccination) for individuals with Down Syndrome (DS, blue circle) and Healthy Control (HC, red square) vaccinated with either the BNT162b2 (solid) or ChAdOx1 (empty) vaccine. Threshold of seronegativity was determined at 14.3 BAU/ml (horizontal dotted line).

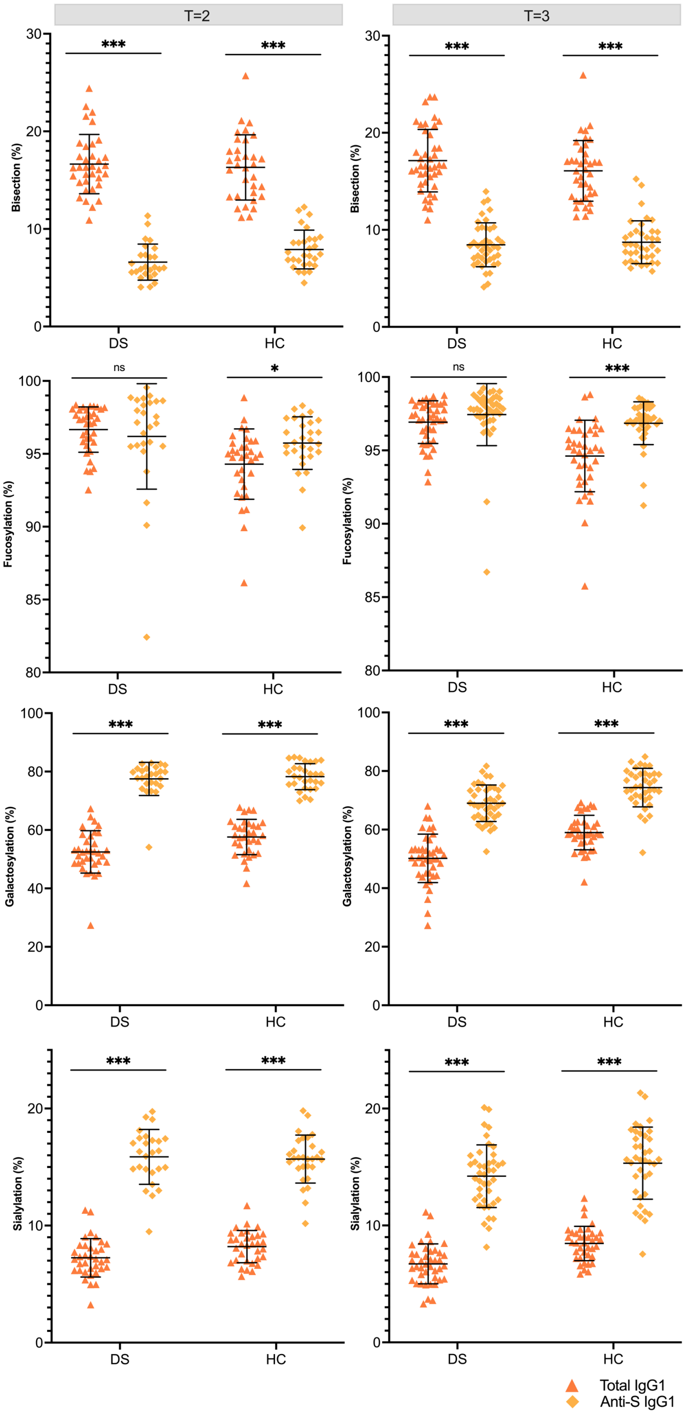

Supplemental Figure 5 – Comparing total and anti-S IgG1 Fc Glycosylation

Total (orange, triangle) and anti-S (yellow, diamond) IgG1 Fc glycosylation at T=2 (±28 days after 2nd vaccination, left) and T=3 (±28 days after 2nd vaccination, right). Levels were compared using a paired-samples T test or Wilcoxon signed-rank test, depending on normality with ns; not significant, *; *p*-value <0.05, **; *p*-value < 0.01, ***; *p*-value < 0.001.

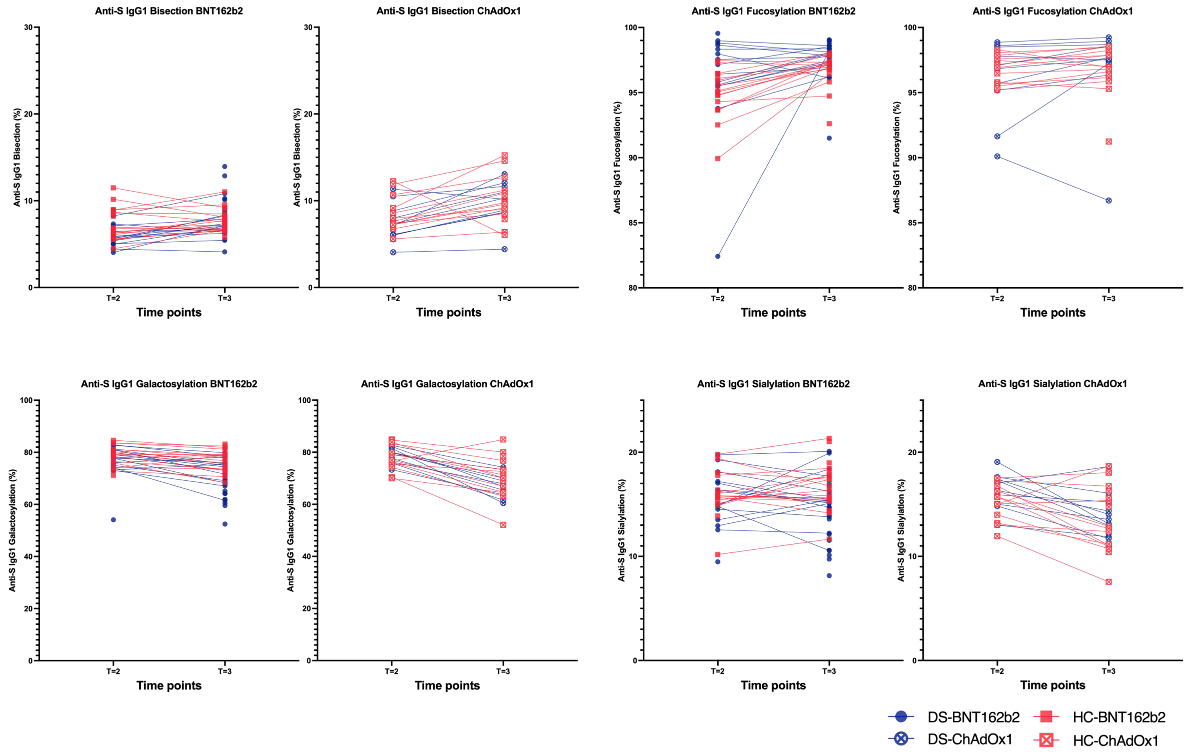

Supplemental Figure 6 – Anti-S IgG1 Fc Glycosylation in time

Anti-S IgG1 Fc glycosylation in time at T=1 (before vaccination), T=2 (±28 days after 2nd vaccination) and T=3 (±28 days after 2nd vaccination) for individuals with Down Syndrome (DS, blue circle) and Healthy Control (HC, red square) vaccinated with either the BNT162b2 (solid) or ChAdOx1 (empty) vaccine.

Supplemental table 1 - IgG1 glycopeptides included in the final analyte list

Glycan composition	Alternative nomenclature	$[M+2H]^{2+}$	$[M+3H]^{3+}$	Proposed structure
**H3N4F1**	G0F	1317.527	878.687	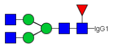
**H4N4**	G1	1325.524	884.018	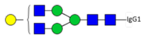
**H4N4F1**	G1F	1398.553	932.704	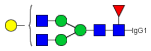
**H5N4**	G2	1406.550	938.036	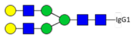
**H5N4F1**	G2F	1479.579	986.722	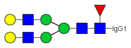
**H5N4S1**	G2S	1552.098155	1035.068	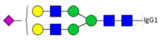
**H5N5F1S1**	G2FNS	1726.667	1151.447	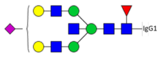
**H5N4F1S2**	G2FS2	1770.675	1180.786	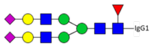
**H4N5F1**	G1FN	1000.398	1500.093	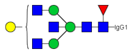
**H4N4F1S1**	G1FS	1029.736224	1544.101	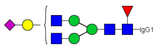
**H5N5F1**	G2FN	1054.415	1581.119	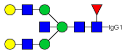
**H5N4F1S1**	G2FS	1083.753832	1625.127	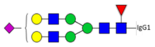

Supplemental table 2 - Description and calculation of IgG1 glycosylation traits.
H: hexose, N: *N*-acetylhexosamine, F: fucose, S: *N*-acetylneuraminic (sialic) acid

	Description	Formula
**IgG1 bisection**	*N*-glycans carrying a bisected *N*-acetylglucoseamine	(H5N5F1S1 + H4N5F1 + H5N5F1) / sum of all IgG1 glycopeptides
**IgG1 galactosylation**	*N*-glycans carrying galactose(s)	(1/2 * (H4N4 + H4N4F1 + H4N5F1 + H4N4F1S1) + 2/2 * (H5N4 + H5N4F1 + H5N4S1 + H5N5F1S1 + H5N4F1S2 + H5N5F1 + H5N4F1S1)) / sum of all glycopeptides
**IgG1 sialylation**	*N*-glycans carrying *N*-acetylneuraminic (sialic) acid(s)	(1/2 * (H5N4S1 + H5N5F1S1 + H4N4F1S1) + 2/2 * H5N4F1S2) / sum of all IgG1 glycopeptides
**IgG1 fucosylation**	*N*-glycans carrying a core fucose	(H3N4F1 + H4N4F1 + H5N4F1 + H5N5F1S1 + H5N4F1S2 + H4N5F1 + H4N4F1S1 + H5N5F1S1 + H5N4F1S1) / sum of all IgG1 glycopeptides
