# Supplementary material for: IgG1 glycosylation highlights premature aging in Down syndrome
Source: Aging Cell. 2024 Apr 15;23(7):e14167. doi: 10.1111/acel.14167 (PMC11258452; doi:10.1111/acel.14167)
Supplement: Supplementary file 2 — Appendix S2 [file ACEL-23-e14167-s001.pdf]

IgG glycosylation highlights premature aging in Down Syndrome

PRIDE study group collaborators are:

Antonia M.W. Coppus[1], Esther de Vries[2,3], Michel E. Weijerman[4], Regina Lamberts[5] and Gert de Graaf[5]

[1] Department for Primary and Community Care, Radboud University Medical Center, Nijmegen, The Netherlands

[2] Tranzo, Tilburg School of Social and Behavioral Sciences, Tilburg University, Tilburg, The Netherlands

[3] Jeroen Bosch Academy Research, Jeroen Bosch Hospital, ‘s-Hertogenbosch, The Netherlands

[5] Department of Pediatrics, Alrijne Hospital, Leiderdorp, The Netherlands

[5] Foundation ‘Stichting Down Syndroom’, Meppel, The Netherlands
